# Long Frontal Projections Help *Battus philenor* (Lepidoptera: Papilionidae) Larvae Find Host Plants

**DOI:** 10.1371/journal.pone.0131596

**Published:** 2015-07-29

**Authors:** Ikuo Kandori, Kazuko Tsuchihara, Taichi A. Suzuki, Tomoyuki Yokoi, Daniel R. Papaj

**Affiliations:** 1 Laboratory of Entomology, Faculty of Agriculture, Kinki University, Nara, Japan; 2 Forestry and Forest Products Research Institute, Tsukuba, Japan; 3 Department of Integrative Biology, University of California, Berkeley, United States of America; 4 Laboratory of Conservation Ecology, Faculty of Life and Environmental Sciences, University of Tsukuba, Tsukuba, Japan; 5 Department of Ecology and Evolutionary Biology, University of Arizona, Tucson, United States of America; Institute of Plant Physiology and Ecology, CHINA

## Abstract

Animals sometimes develop conspicuous projections on or near their heads as, e.g., weaponry, burrowing or digging tools, and probes to search for resources. The frontal projections that insects generally use to locate and assess resources are segmented appendages, including antennae, maxillary palps, and labial palps. There is no evidence to date that arthropods, including insects, use projections other than true segmental appendages to locate food. In this regard, it is noteworthy that some butterfly larvae possess a pair of long antenna-like projections on or near their heads. To date, the function of these projections has not been established. Larvae of pipevine swallowtail butterflies *Battus philenor* (Papilionidae) have a pair of long frontal fleshy projections that, like insect antennae generally, can be actively moved. In this study, we evaluated the possible function of this pair of long moveable frontal projections. In laboratory assays, both frontal projections and lateral ocelli were shown to increase the frequency with which search larvae found plants. The frontal projections increased finding of host and non-host plants equally, suggesting that frontal projections do not detect host-specific chemical cues. Detailed SEM study showed that putative mechanosensillae are distributed all around the frontal as well as other projections. Taken together, our findings suggest that the frontal projections and associated mechanosensillae act as vertical object detectors to obtain tactile information that, together with visual information from lateral ocelli and presumably chemical information from antennae and mouthparts, help larvae to find host plants. Field observations indicate that host plants are small and scattered in southern Arizona locations. Larvae must therefore find multiple host plants to complete development and face significant challenges in doing so. The frontal projections may thus be an adaptation for finding a scarce resource before starving to death. This is the first evidence that arthropods use projections other than true segmental appendages such as antennae, mouthparts and legs, to locate food resources.

## Introduction

Animals sometimes bear conspicuous projections on or near their heads, such as horns, antlers, tusks, enlarged mandibles, barbels, antennae, or spines. These projections can be divided into four groups according to their function. First, adult males with such structures may be favoured by conspecific females or may use them in intra-sexual contests for mates. Examples include horns and antlers of ungulates, and tusks of elephants and walruses in mammals [1–4], horns and enlarged mandibles of some beetles [5–8], and eye stalks of flies in insects [9,10]. Second, these structures may be used as anti-predator weaponry. Examples include horns of horned lizards, and female bovids, and spines on the pronotum of the pygmy grasshopper *Criotettix japonicus* [11–13]. Third, protruding tusk-like teeth or horns may be used to burrow or dig by animals such as naked mole rats and the sand-living anthicid beetle *Mecynotarsus tenuipes* [14,15]. Fourth, some projections have many mechanical and chemical sensors on their surface [16–19] and may be used to locate resources such as foods, host plants, and mates [20–22]. Examples include barbels in fish and antennae in insects and other arthropods such as centipedes, millipedes, macrurans, hermit crabs, and pill bugs. In addition to antennae, insects also use maxillary palps and labial palps to detect and assess foods. However, to date, there is no evidence that arthropods, including insects, use projections except antennae and other segmental appendages to locate and assess food.

In this respect, it is interesting to note that some butterfly larvae such as *Sasakia charonda*, *Cyrestis thyodamas*, *Parantica sita*, *Sericinus montela*, *Dichorragia nesimachus*, *Idea leuconoe*, *Araschnia burejana*, *Melanitis phedima*, and *Ariadne ariadne* possess a pair of long frontal projections on or near their heads. The projections look like antennae but are not; their actual antennae are small, short, three-segmented structures found close to their mouthparts [23,24]. The function of the frontal projections is not known. Larvae of pipevine swallowtail butterfies *Battus philenor* L. (Papilionidae) also have a pair of the long frontal projections. These larvae have many elastic, fleshy and non-segmented projections on their dorsal surface. The most forward pair of projections adjacent to the head capsule (hereafter we say “frontal projections”) are particularly long. Furthermore, unlike the more posterior projections, the larva can actively move each frontal projection (S1 Movie). It has yet to be established why these paired frontal projections are so long and why the larva actively moves only this pair of projections. Here we propose a hypothesis that larvae use the frontal projections to locate host plants. We predict that frontal projections of the larvae may enhance detectability or searching efficiency of host plants, when they search for the next host plant.

In this study, we specifically addressed four questions to confirm this prediction: (1) Do frontal projections enhance finding of host plants? (2) Do frontal projections allow the larva to distinguish between host and non-host plants? (3) What type(s) of sensillae are borne on the frontal projections? (4) What properties of the host plants of pipevine swallowtail larvae favour use of frontal projections to find food? To our knowledge, we provide the first evidence that arthropods use conspicuous projections, other than true segmental appendages such as antennae, mouthparts and legs, to gather information about their food resources.

## Materials and Methods

### (a) Study species

*Battus philenor* is distributed throughout much of the U.S., all of Mexico and much of Central America [25]. The butterfly is an extreme specialist. Larval host plants for members of the genus *Battus* belong exclusively to the genus *Aristolochia* (Family Aristolochiaceae). In southern Arizona, *B*. *philenor* adults are common between late March and early September. The local host species, *Aristolochia watsonii*, is a small perennial, deciduous, recumbent vine with multiple stems which is abundant in washes and bordering areas. Larvae occur in a black form and a red form [26,27]; in southern Arizona, the red form is more common. *B*. *philenor* larvae of both color forms have many projections located medio-laterally along each side. The paired frontal projections, adjacent to the head capsule, are distinctively long, especially in the fourth and the last (fifth) instar (S1 Fig). The larva can actively move this pair of projections but not the other ones (S1 Movie).

In our study, eggs and larvae were obtained from 25 hectares of mesquite grassland on the Santa Rita Experimental Range (SRER) in Pima County, Arizona, USA (318 47.0490 N; 1108 49.5240 W), which is managed by the University of Arizona. Eggs were also obtained from gardens on the university campus where wild females oviposited on cultivated *A*. *fimbriata*. Larvae were raised in the laboratory at room temperature (*ca* 26°C) under natural photoperiod (*ca* 14: 10 h light: dark). Field measurements were also made at the SRER field site, under the auspices of a permit granted by the University of Arizona to one of the co-authors (Papaj).

### (b) The success of finding host plants by searching larvae

To address the first question of whether frontal projections facilitate location of host plants, we performed 2 treatments for each fifth-instar larva. The first treatment involved removing frontal projections. We found that, when we bound the base of frontal projections with a fine thread during the inactive period in the late fourth before molting to the fifth instar, larvae successfully molted and lost frontal projections without any obvious loss of hemolymph (S2 Fig). Measurements revealed no significant difference in walking speed between the larvae with versus without frontal projections (see Results). We therefore assumed that removing projections by this method did not decrease larval movement in a way that might reduce host plant searching efficiency. The second treatment involved blinding the caterpillars. This treatment was included because pre-test observations suggested that visual input to the lateral ocelli was used when larvae search for host plants. Larvae were blinded by painting the entire head capsule, including the lateral ocelli, excluding antennae and mouthparts, with white water-soluble paint followed by green paint. We painted twice for each larva to ensure that vision was abolished. We changed colors to ensure that the second coat covered the first coat. There was no significant difference in walking speed between vison-capable versus blind larvae (see Results).

**Fig 1:**
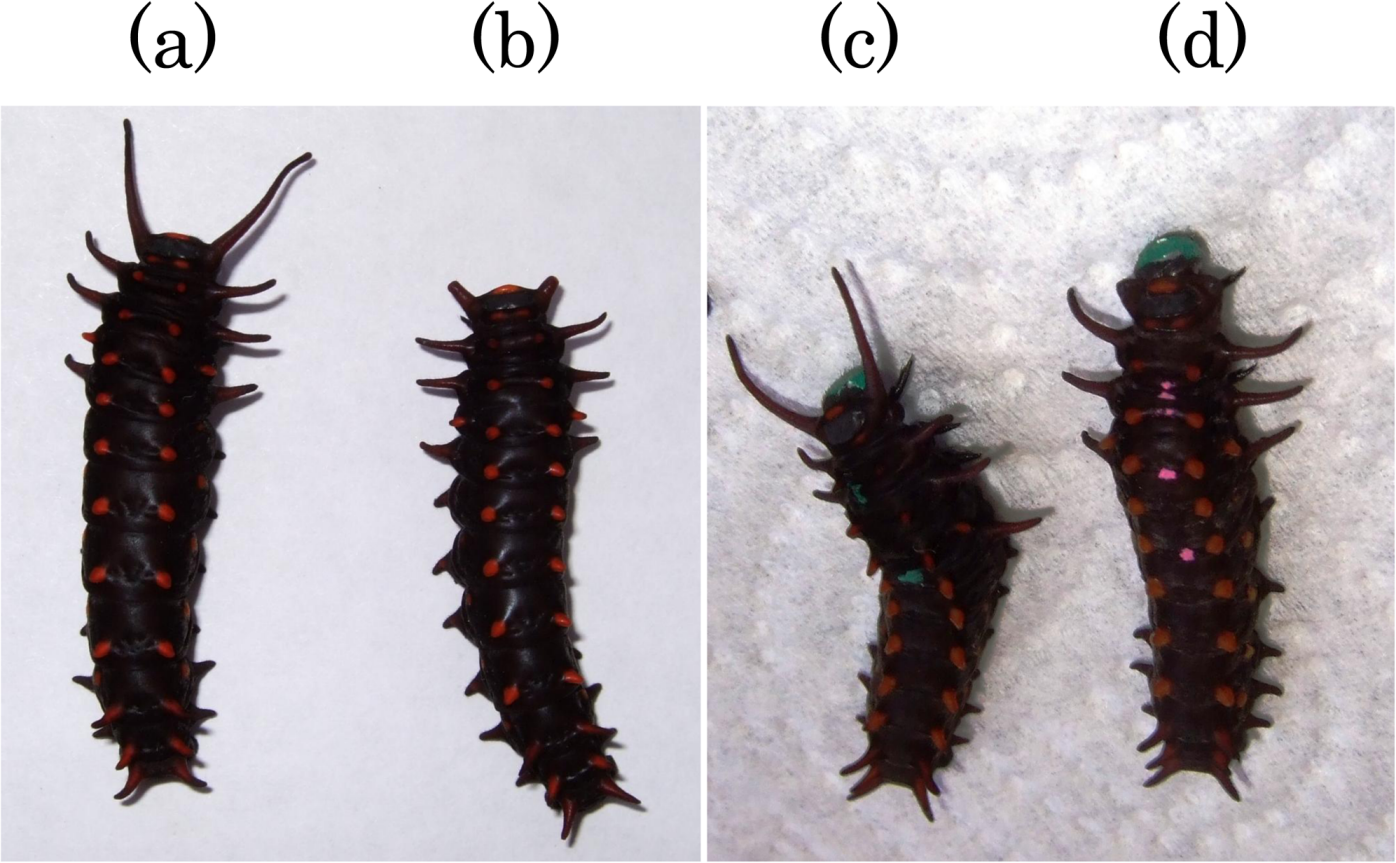
Four types of larvae used in the experiment investigating the success of finding host plants. (a) vision-capable larva with frontal projections intact (normal larvae), (b) vision-capable larva lacking frontal projections, (c) blind larva with frontal projections intact, (d) and blind larva lacking frontal projections.

Summarizing, we produced four types of larvae with different combinations of two treatments, i.e., (a) vison-capable larvae with frontal projections intact (normal larvae), (b) vision-capable larvae lacking frontal projections, (c) blind larvae with frontal projections intact, and (d) blind larvae lacking frontal projections (Fig 1).

To quantify the finding of host plants by searching larvae, we first constructed a wooden stick walkway for the larva. This walkway was 20cm long and 0.4cm in diameter. Each end of the walkway was mounted on and fixed with a short wooden stick (4.5cm long and 0.6cm in diameter) which was set at right angles to the stick walkway. This walkway setup was placed on a table in the laboratory. Next we placed a fresh 3cm-stem of host plant (*A*. *fimbriata*) on each side of the walkway, 1.5 or 2cm apart from the walkway.

**Fig 2:**
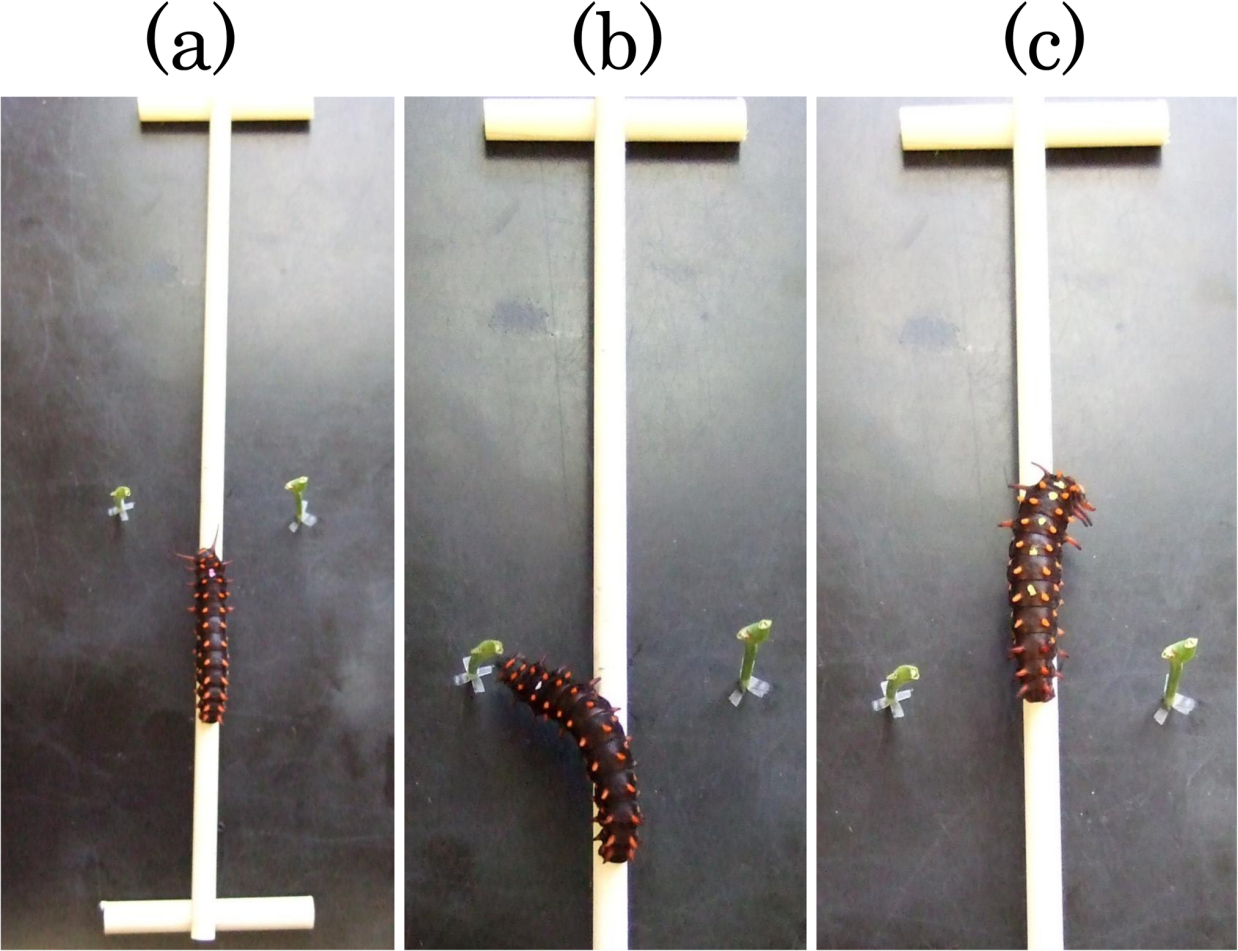
Photos of the experiment to investigate the success of finding host plants by searching larvae. (a) A larva walking along the walkway before reaching the nearest point to the host plant stems. (b) A larva that successfully found the host plant stem. (c) A larva that failed to find the stems, approaching the opposite end of the walkway.

We made test larvae hungry by maintaining them without host plant for one day. Then we allowed each hungry larva to walk along the stick walkway and to search for host plant (Fig 2). Larva readily walked along the top of the stick, approximately 1 cm above the table, and never walked on the underside of the stick. They seldom moved from the walkway to the table (those few instances when such movement occurred during the experiment were not counted as trials). As larvae walked along the walkway, they frequently moved their heads from side to side to search for host plant. When they successfully found the host plant stem, they invariably touched it with their antennae and mouthparts and began to chew. We recorded the larva as ‘finding a stem’ if it touched a stem with antennae and mouthparts. We recorded the larva as ‘failing to find a stem’ if it reached the opposite end of the walkway without touching either stem with antennae and mouthparts. If the larvae had frontal projections, they actively moved frontal projections during walking and they often touched the stem with frontal projections before touching it with antennae and mouthparts. We tested each larva 10 times (10 trials) continuously without interval between trials. Usually, the duration of one trail was less than 15 seconds (see Results). We calculated each larva’s success in finding hosts as the proportion of the 10 trials in which they found a host plant stem. In this experiment we used fifth-instar larvae, specifically larvae that were three to five days after the fourth molt. The experiment was conducted in the laboratory at room temperature under natural sunlight on June 9 –August 11, 2010.

To confirm that there was no difference in walking (searching) speeds that might affect host plant searching efficiency among the four types of larvae, an additional experiment was conducted using the same experimental protocol as above except that the host plant stem was removed from the middle of the walkway. A hungry larva was allowed to walk from one end to the opposite end of the walkway and the time required to traverse the walkway was recorded.

#### Statistical analysis

We used a fixed effects analysis of variance [ANOVA; general linear model (GLM) with type III sums of squares] to test for effects on the arcsine square-root transformed proportion of instances in which host plants were found by larvae, in which independent factors were distance (whether host plant stems were 1.5 or 2.0 cm apart from the walkway), eye treatment (whether larva were vision-capable or blind), projection treatment (whether larva had a pair of frontal projections or not) and their interactions. We also used a fixed effects ANOVA (GLM) to test for effects on walking speed (log+1 transformed) of searching larvae, in which independent factors were eye treatment, projection treatment and their interactions. IBM SPSS statistics 22 [28] was used for all statistical analysis.

### (c) Discovery of host and non-host plants by vision-capable searching larvae

To address the second question of whether frontal projections allow the larva to distinguish between host and non-host plants, we conducted a behavioural assay similar to the above assay, using the same walkway. Here we used only vision-capable larvae with frontal projections either intact or lacking. The plant stems were set 2cm away from the walkway. In this assay, we used either of 2 types of plant stems, i.e., those of host plants (*A*. *fimbriata*) or non-host plants (*Eragrostis lehmanniana*). We selected *E*. *lehmanniana* as a representative non-host plant because it is the dominant non-host plant species at the SRER field site. We allowed each hungry larva to traverse the walkway and search for plant stems. We tested each larva 10 times using the same type of plant stems, i.e., the plant stem type of the right and the left side of the walkway was the same and one larva was tested for only one type of plant stems during 10 trials. That is, half of the larvae with frontal projections either intact or lacking were assayed for only host plant stems and the other half were assayed for only non-host plant stems. We then calculated the proportion of the 10 trials in which they found a plant stem. Other experimental conditions such as hunger level and age of larvae and plant stem size were the same as in the previous assay. The experiment was conducted in the laboratory at room temperature under natural sunlight on August 17–24, 2010.

#### Statistical analysis

We used a fixed effects ANOVA (GLM) with type I sums of squares to test for effects on the arcsine square-root transformed proportion of instances in which plant stems were found by larvae, in which independent factors were projection treatment (whether larva had a pair of frontal projections or not), plant (whether plant stems were those of host or non-host), and their interactions.

### (d) Observation of sensilla on the projections by SEM

To address the third question of what type(s) of sensillae caterpillars have on frontal projections, a scanning electron microscope (SEM) study was conducted on frontal projections as well as the other projections on the larval body surface. Fifth-instar larvae of *B*. *philenor* were collected from a garden on the university campus. Twenty caterpillars were checked under a stereomicroscope. The whole bodies of caterpillars were fixed in 2.5% glutaraldehyde buffered in phosphate, pH 7.2, for 12 h, repeatedly rinsed in the same buffer. The specimens were dehydrated by using ethanol gradients, followed by critical-point drying in a CO2 (JCPD-3, JOEL,Tokyo, Japan). The specimens were mounted on aluminum stubs, sputter-coated with platinum (JFC-1100, JOEL, Tokyo, Japan), observed and photographed in a Hitachi S-4800 of the Field Emission-Scanning Electron Microscope (FE-SEM).

### (e) The distance between individual *A*. *watsonii* plants

To address the fourth question of what host properties might favour use of frontal projections in host finding, we conducted a field study of their local host plant, *A*. *watsonii*. We first measured the straight-line distance to the nearest conspecific for 51 individual *A*. *watsonii* plants. We measured the distance between the nearest edges of two individual plants, which is equivalent to the minimum distance that *B*. *philenor* larvae must walk between the two plants. Measurements were conducted on July 3 and 4, 2010.

### (f) The mass of *A*. *watsonii* foliage that *B*. *philenor* larvae eat to complete their development relative to the mass of foliage of individual *A*. *watsonii* plants

To further address the fourth question of what host properties might favour use of frontal projections, we estimated the mass of *A*. *watsonii* foliage eaten by *B*. *philenor* larva throughout their development and compared this estimate to an average of the mass of individual plants in the field. Estimates of mass eaten was done in two steps. In the first step, a fourth- or fifth-instar larva was kept in a transparent plastic cup without food for one day such that guts would be evacuated. The fourth- or fifth-instar larva was then allowed to eat 0.5 or 1.0 g of fresh *A*. *watsonii* completely, respectively, and its excrement, or frass, was collected. The frass was dried in a drying oven at *ca* 55°C for one day and weighed with an analytical balance. This step made it possible to estimate the weight conversion rate from fresh host plant eaten to dry frass. This value was assumed to be a constant across different instars and for different individuals.

In the second step, we reared *B*. *philenor* larvae of different instars individually on fresh *A*. *watsonii* in a transparent plastic cup, and collected their frass during each larval instar, which was then dried and weighed with the same methods mentioned above. These data, in combination with the weight conversion rate estimated above, allowed us to estimate how much fresh host material *B*. *philenor* larva ate during each instar. Then we summed these estimates for all instars to calculate total amount of host materials consumed throughout its larval development. We used 11, 14, 12, 13 and 12 individual larvae for the first, second, third, fourth and fifth (last) instar, respectively. The experiment was conducted on July 5–29, 2010.

We also weighed the above-ground foliage of 51 *A*. *watsonii* plants collected in the field. To do this, we first cut all of a given *A*. *watsonii* plant above the ground at the SRER field site. The foliage for each plant was placed in its own zippered plastic bag and the bag placed in a portable ice chest. The foliage was weighed using an electronic balance in the laboratory at the University of Arizona within 3 hours after cutting. The experiment was conducted on July 3 and 4, 2010.

## Results

### The success of finding host plants by searching larvae

**Fig 3:**
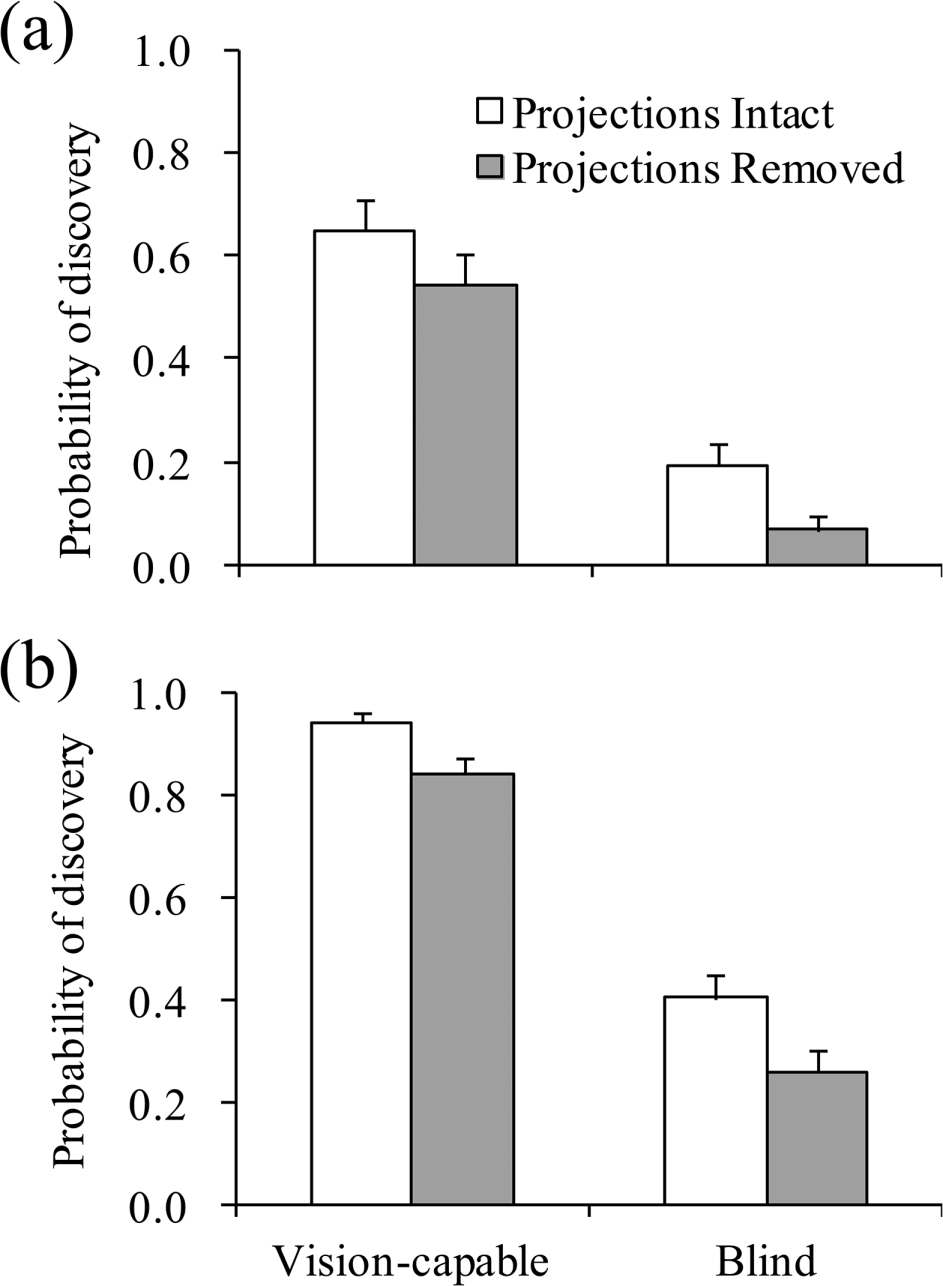
Success (mean + S.E.) of finding host plant stems by searching larvae. Host plant stems were set (a) 2cm and (b) 1.5cm away from the walk way. Larvae were assigned to a combination of 2 treatments. The first treatment related to the pair of frontal projections (intact versus removed). The second treatment related to the eyes (vision-capable versus blind). N = 18 (numbers of larvae used) for each bar.

Success in finding host plant stems by searching larvae was generally higher when the host plants were closer, when the larvae were vision-capable, and when frontal projections were present (Fig 3). Frontal projections increased success in finding host plants by 10–15% when they were compared between the larvae with versus without frontal projections at the same distance to the stem and within the same eye treatment. The ANOVA for discovery rate of the host plant stem indicated that there was a significant effect of distance, eye treatment and projection treatment and a nonsignificant effect of their interaction (Table 1). These results suggest that *B*. *philenor* larvae use both eyes (lateral ocelli) and frontal projections to enhance finding of host plants.

**Table 1:** ANOVA of the discovery rate of the host plant stems by *B*. *philenor* larvae. ‘Distance’ refers to the effect of distance between the walkway and host plant stems (1.5 or 2 cm). ‘Eye’ refers to the effect of blinding larvae. ‘Projection’ refers to the effect of creating larvae lacking frontal projections. Significant p-values are in bold.

Source	df	MS	F	P
Distance	1	4.697	70.082	**0.000**
Eye	1	17.037	254.221	**0.000**
Projection	1	1.353	20.186	**0.000**
Distance×Eye	1	0.061	0.912	0.341
Distance×Projection	1	0.005	0.069	0.794
Eye×Projection	1	0.014	0.214	0.645
Distance×Eye×Projection	1	0.014	0.203	0.653
Error	136	0.067		

Mean ±S.E. of walking speeds (seconds/20cm-walkway) of searching larva were 12.4±1.4 (n = 14), 11.9±1.2 (n = 14), 14.2±2.0 (n = 14), and 12.6±1.7 (n = 14), for vison-capable larvae with frontal projections intact, vision-capable larvae lacking frontal projections, blind larvae with frontal projections intact, and blind larvae lacking frontal projections, respectively. The ANOVA on walking speed indicated that there was no significant effect of eye treatment (F = 0.258, P = 0.613), projection treatment (F = 0.408, P = 0.526) or their interaction (F = 0.060, P = 0.807) on walking speed of searching larvae. The above results suggest that there was no difference in the walking speeds or larval activities that might affect searching efficiency of host plant among the four types of larvae.

### Discovery of host and non-host plants by vision-capable searching larvae

**Fig 4:**
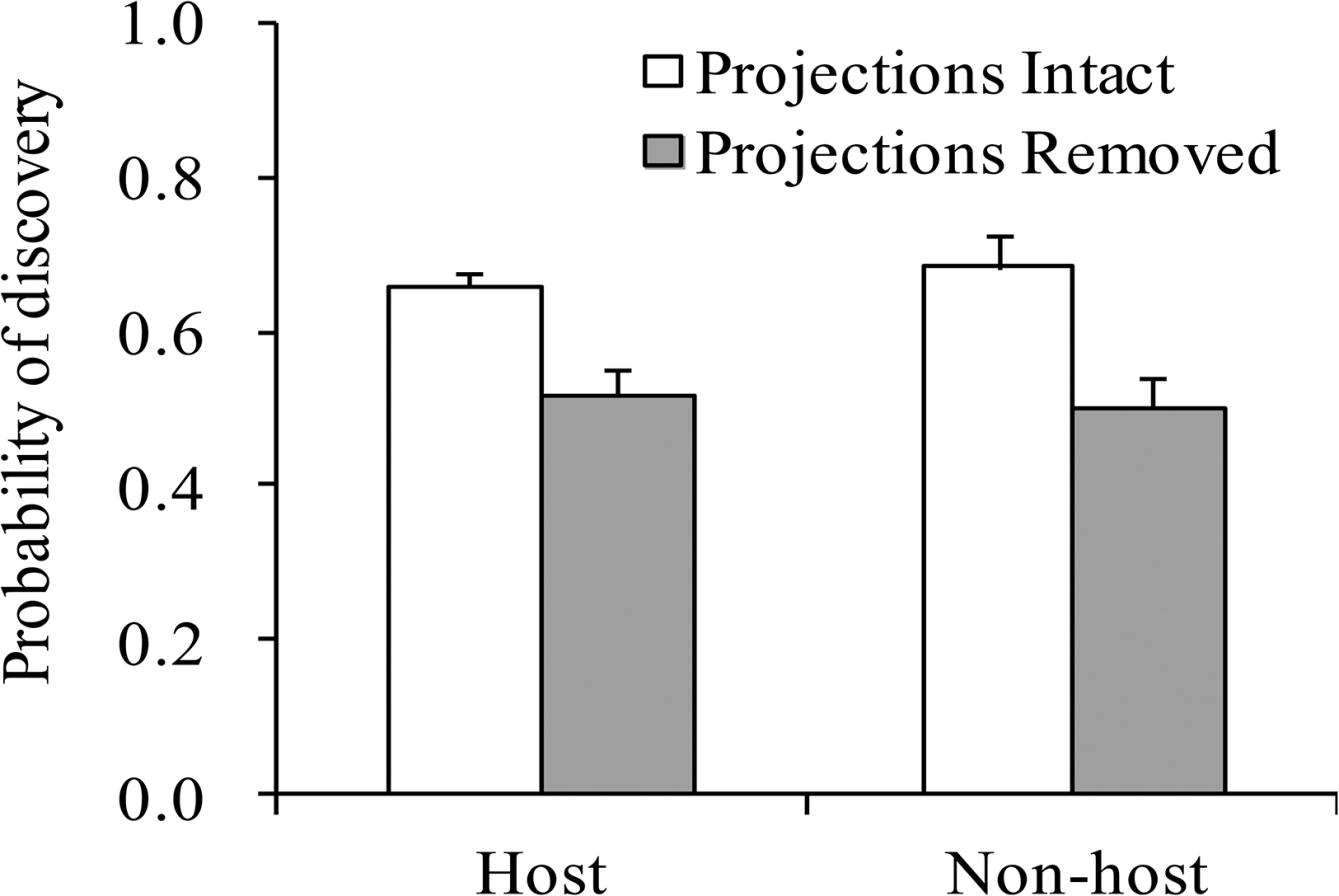
Proportion encounters (mean + S.E.) of host and non-host plant stems by vision-capable searching larvae. Either host (*Aristolochia fimbriata*) or non-host (*Eragrostis lehmanniana*) plant stems were set 2cm away from the walk way. Frontal projections of the larvae were either intact or removed. N = 12 (numbers of larvae used) for each bar.

Vision-capable larvae discovered plant stems more often when frontal projections were present than when they were absent regardless whether plant stems were host or non-host (Fig 4). Larvae with frontal projections intact found the host and non-host plant stems at the same frequency, as did larvae lacking frontal projections. The ANOVA on discovery rate of a plant stem indicated that there was a significant effect of projection treatment, a nonsignificant effect of plant stem type and a nonsignificant interaction (Table 2). Taken together, these results suggest that frontal projections facilitate the discovery of a plant, but do not bias discovery towards host plants.

**Table 2:** ANOVA of the discovery rate of the host and non-host plant stems by vision-capable searching larvae. ‘Projection’ refers to the effect of larvae with frontal projections intact and larvae lacking frontal projections. ‘Plant’ refers to the effect of plant stem type, host plant (*Aristolochia fimbriata*) versus non-host plant (*Eragrostis lehmanniana*). Significant p-values are in bold.

Source	df	MS	F	P
Projection	1	0.569	5.823	**0.020**
Plant	1	0.018	0.188	0.667
Projection×Plant	1	0.021	0.213	0.647
Error	44	0.098		

### Observation of sensillae on the projections by SEM

**Fig 5:**
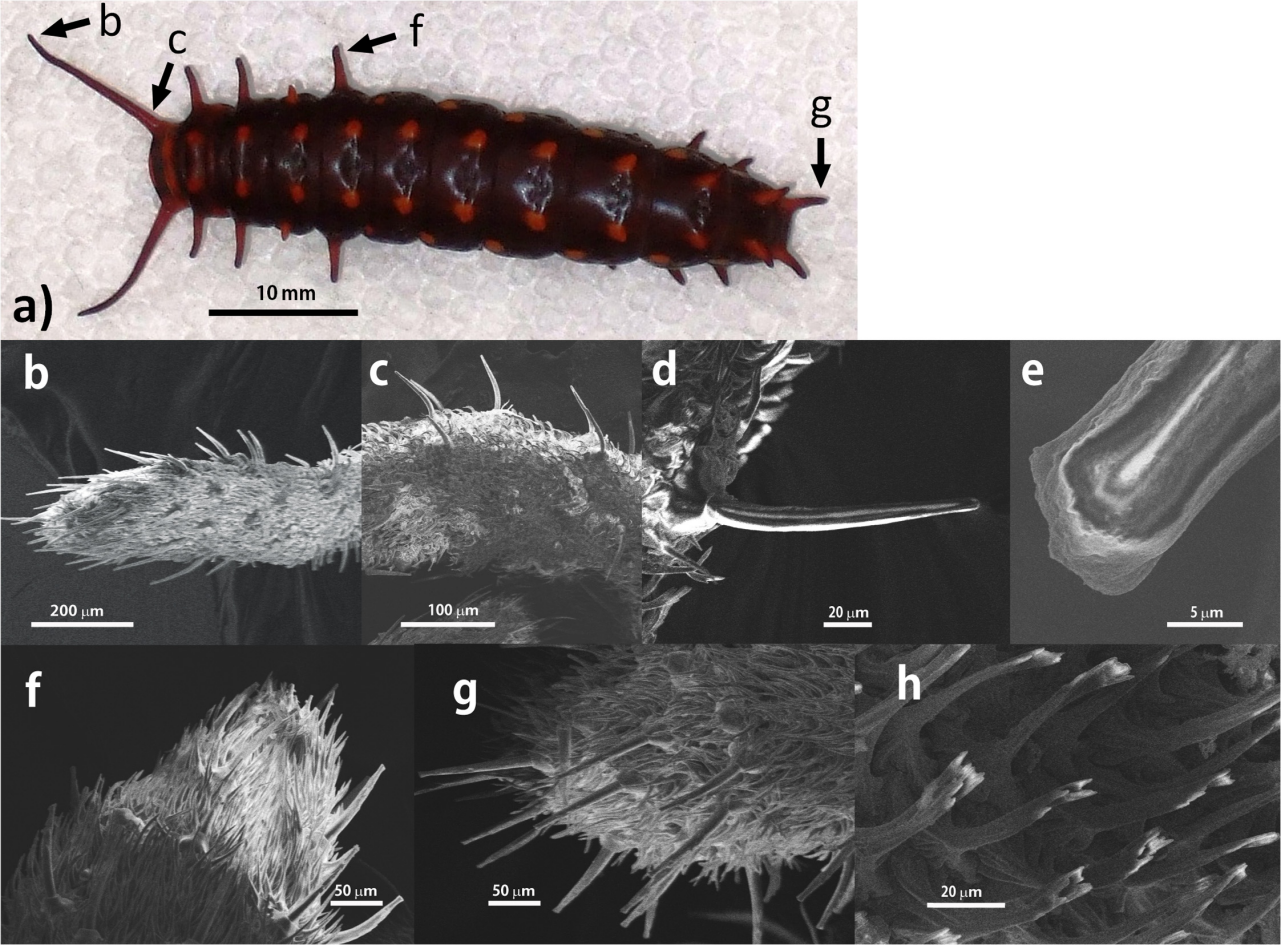
Scanning electron micrographs of the projections, and two types of hairs on the projections. a) Fifth instar larvae. Arrows indicate the view point by SEM. b) The tip of frontal projection. c) The bottom of frontal projection. d) A long type of hair which is longitudinally sectioned. e) Close-up of the tip of a long type of hair which is longitudinally sectioned. f) The tip of the middle projection. g) The tip of the terminal projections. h) Short type of hairs.

We observed numerous hairs on the long frontal projections by the scanning electron microscope (SEM). Hairs on the projections could be classified into two groups based on their length: short type [length: 41.65±1.59 (mean±S.E.) μm, n = 6] and long type (length: 114.65±3.70μm, n = 6; width: 11.74±0.54μm, n = 6; socket width: 45.60±1.47μm, n = 6). The short type are more numerous, and appear to be setae. In contrast, the long type appear to be sensillae. Since the long type of hairs has neither lateral nor terminal pores, they are putative mechanosensillae rather than chemosensillae. There was no obvious difference in the density of mechanosensillae between frontal projections and more distal projections on the larval body (Fig 5). There were 504.33±34.55 (n = 6) mechanosensillae on a single frontal projection. We could not find any putative chemosensillae in the frontal and other projections. These observations are in consistent with the results of the above behavioural assay that frontal projections do not bias discovery towards host plants.

### The distance between individual *A*. *watsonii* plants

**Fig 6:**
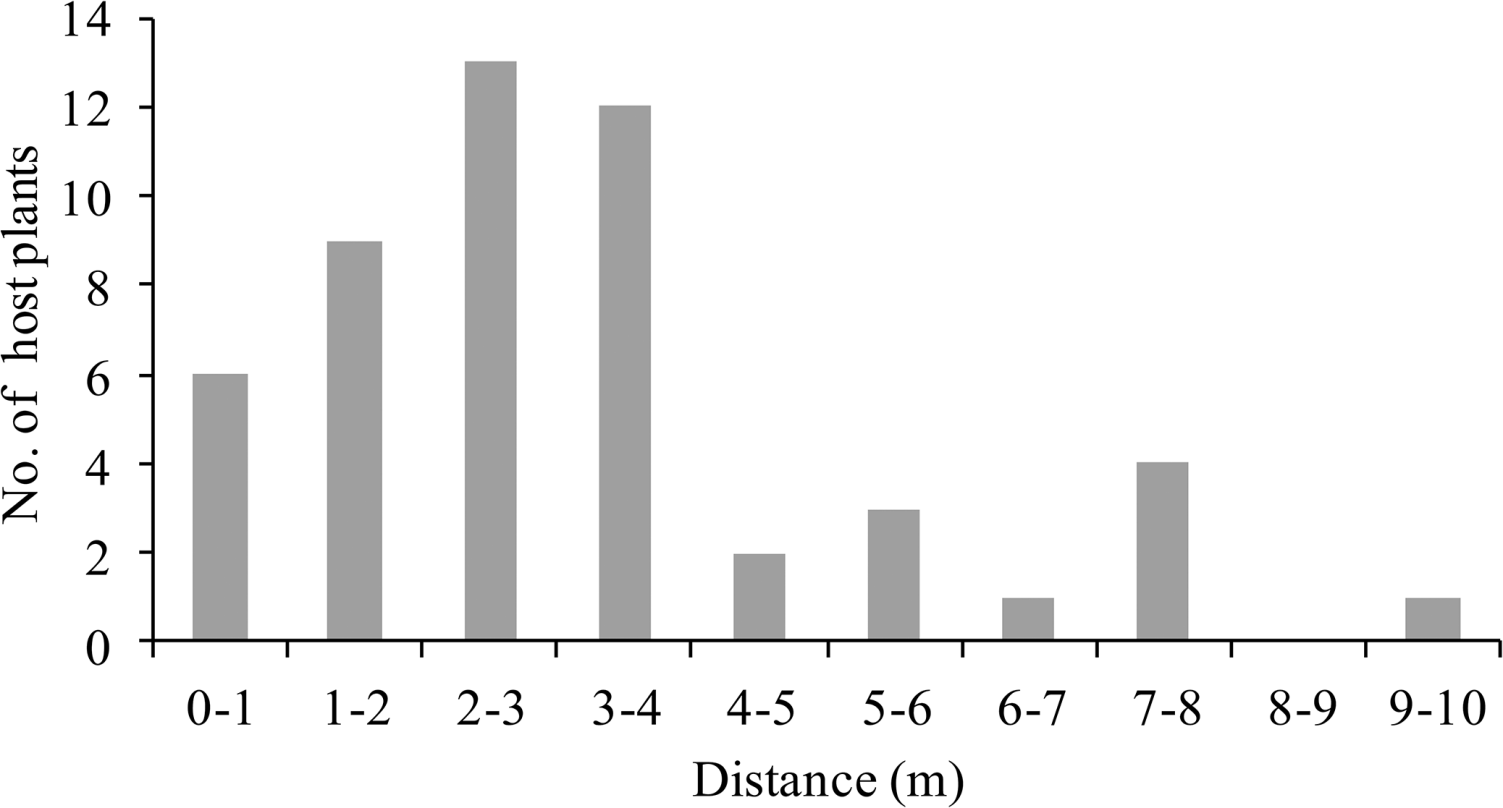
Distance between the nearest conspecifics of *A*. *watsonii* in the field.

In the field, the mean distance between the nearest conspecific of *A*. *watsonii* was 3.00±0.29m (mean±S.E. n = 51) (Fig 6).

### The mass of *A*. *watsonii* foliage that *B*. *philenor* larvae eat to complete their development relative to the mass of foliage of individual *A*. *watsonii* plants

**Fig 7:**
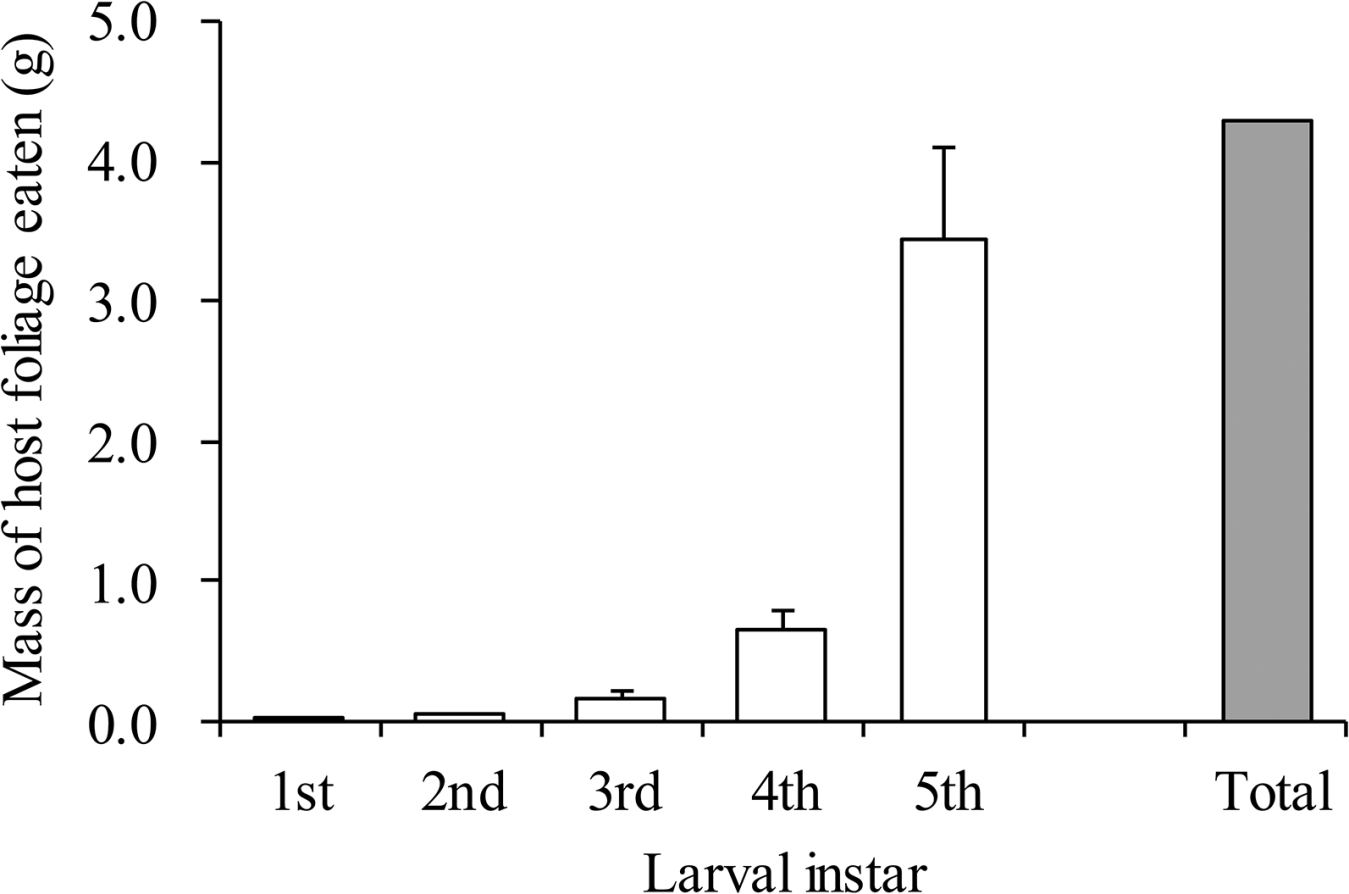
Estimated mass of host plant foliage in fresh weight consumed by *B*. *philenor* larvae. Blank bars indicate consumption during each instar (mean + S.D.) and a filled bar during the total larval period (sum of means for 1–5 instars).

In the first step, we got the weight conversion rate of 0.159 ±0.005 (n = 29) from fresh host plant eaten to dry frass. By using this value in the second step, a single larva of first, second, third, fourth, and fifth instar was estimated to eat 0.007 ±0.001 (n = 11), 0.034 ±0.006 (n = 14), 0.148 ±0.021 (n = 12), 0.645 ±0.042 (n = 13), and 3.458±0.185g (n = 12), respectively. As a total, 4.292g of host foliage in fresh weight was needed to complete its development (Fig 7). A fifth-instar larva ate 80.2% of the total weight eaten throughout larval development.

**Fig 8:**
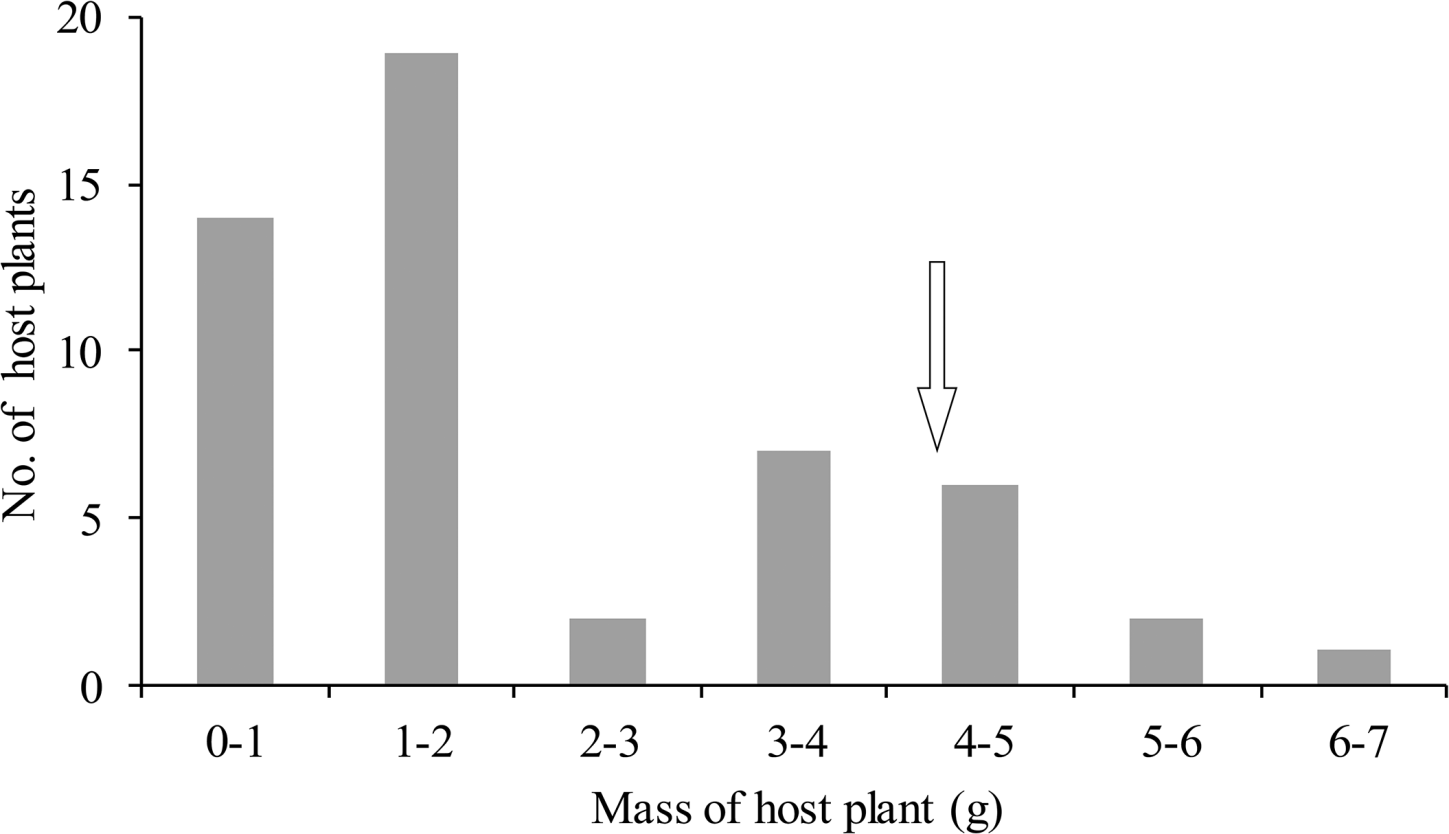
Mass of *A*. *watsonii* foliage collected from the field. Mean±S.E. = 2.13±0.23 (g), n = 51. Arrow indicates estimated mass of host plants eaten by *B*. *philenor* throughout larval development (see Fig 7).

In the field, the mean fresh weight of foliage for an *A*. *watsonii* plant was 2.125±0.226g (n = 51) (Fig 8), which was significantly smaller than the fresh weight of *A*. *watsonii* that *B*. *philenor* larvae eat during the fifth instar (Mann–Whitney U test; P = 0.008). More than 85% (44/51) of *A*. *watsonii* plants at the field site weighed less than the amount of food that a single *B*. *philenor* larva is estimated to eat to complete its development.

Our results, taken together, indicate that, in the field, *A*. *watsonii* plants are so small and scattered that *B*. *philenor* larvae must 1) use more than one host plant to complete development and 2) travel meters to find the next plant.

## Discussion

In previous studies, insects have been shown to use the extraordinary projections on their body surface as weaponry for intra-sexual contests [6–8,29], inducements for inter-sexual attraction [9,10], weaponry for anti-predator defence [11,30], and burrowing or digging tools [15]. Our study provides the first evidence that arthropods use projections other than true segmental appendages such as antennae, mouthparts and legs, to search for food.

Our findings demonstrate that, although frontal projections do not allow the *B*. *philenor* larvae to distinguish between host and non-host plants, this pair of projections enhances finding host plants. Because the actual antennae are very short, these projections should allow a larva to increase the width of its search path as it wanders in search of a host plant. The larva broadens the search path still further by moving its head region (and, along with it, the projections) from side to side, as it walks. Additionally, the larvae actively move the projections themselves, which probably facilitates sampling within the widened search path. It is noteworthy that the other shorter but presumptively serially homologous projections along the caterpillar’s body do not move in this way (S1 Movie).

Our results suggest that the improvement in searching area afforded by frontal projections could be especially beneficial in locations, such as southern Arizona, where host plants are meters away from each other and where individual host plants are too small to allow a larva to complete development. Consistent with this explanation is the observation that frontal projections become disproportionately long relative to larval body length in the fourth and the last (fifth) instar (S1 Fig). It is in these later instars that large amounts of host foliage are consumed, forcing larvae to leave their natal plant and find at least one other host plant on which to complete development. In the field, we sometimes found multiple larvae in one host plant (I. Kandori, unpubl. obs.). This phenomenon would accelerate food deprivation and migration of the larvae.

Our findings also indicated that frontal projections facilitate finding of host plants by both vision-capable and blind larvae (Fig 3, Table 1). Thus, frontal projections would be useful in finding host plants both during the day and the night. Indeed, 24-hour observation recorded by infrared video camera in the experimental room showed that *B*. *philenor* larvae eat and walk at night even though their activities become lower during the night than during the day (I. Kandori, unpubl. obs.). However visual information from lateral ocelli may be more important than tactile information from frontal projection in searching host plants. This is because lateral ocelli increased success in finding host plant by 46–58% whereas frontal projections increased by 10–15% when they were compared between the larvae with the same condition except eye or projection treatment (Fig 3).

Results of our latter behavioural assay indicate that frontal projections do not allow a larva to distinguish between host and non-host plants (Fig 4, Table 2), suggesting that frontal projections seem neither to detect host plant olfactory cues from a short distance nor gustatory cues by touching the plant with the projections. The results support the inference that the sensillae that we found on frontal projections are not chemosensillae but mechanosensillae. These results also suggest that, like frontal projections, lateral ocelli and antennae do not allow the larva to distinguish between host and non-host plants even from a short distance, and that the larvae can identify host plants as such only after touching the plant with antennae and mouthparts.

In our behavioural assays, we used the normal larvae with frontal projections intact as a control against the larvae lacking frontal projections. We supposed that vitality of the two types of larvae was not different, because removing projections by the methods that we adopted in this study did not lead to any obvious loss of hemolymph, and because the walking speed of the two types of larvae were not significantly different (see Results). It remains possible that removing projections might affect some feature of searching behavior other than walking speed that might reduce host plant searching efficiency. In the future, we plan to test the success of finding host plants for the larvae in which two projections soon behind frontal projections were tied off as a control against the larvae lacking frontal projections. Similarly, our hypothesis on the role of long frontal projections in host plant seeking was only tested in the specific context of the laboratory experiment. In the future, we hope to test this hypothesis in the field.

Long frontal projections are not restricted to *B*. *philenor*. All species in the genus *Battus* for which we have evidence, including *B*. *polydamas*, *B*. *belus*, and *B*. *ingenuus*, have them in their last instar [31]. In contrast, members of all Troidine genera related to *Battus* have short projections distributed along the body but none, to our knowledge, have elongated frontal projections. Taken together, these observations lead us to propose that long frontal projections evolved in the ancestor of the genus *Battus* and are derived from short frontal projections homologous to those found in Troidine relatives. It would be meaningful to know if the long frontal projections in other members of the genus *Battus* are used in location of host plants. If so, it would be interesting to know in turn if members of the genus consistently face host-finding challenges that are more severe than those of other Troidine genera.

The literature on lepidopteran larval adaptations for finding host plants is scant in contrast to the literature on strategies used by the adult female in finding oviposition sites [26,32–38]. Yet caterpillars commonly have cause to locate host plants. For example, some caterpillars, including *Battus philenor* [39], engage in thermoregulatory behavior in which they move off the host and seek a cooler thermal refuge. The caterpillars must then locate the same or new host plant when extreme temperatures abate. Similarly, some butterflies and moths diapause as caterpillars to overwinter or to live through hot dry summer; in some cases [40–42], the caterpillars leave their host plants, which may be senescing, seek refuge in rock crevices, under plant debris, or on other non-host plants, and seek host foliage anew after they break diapause. It has also been shown that certain caterpillars, notably arctiid species, compulsively mix their diet so as to self-medicate themselves against parasitoids [43–45] such caterpillars necessarily must move from one host plant to another in order to consume foliage of multiple plant species. Caterpillars have been reported to respond to attacks by natural enemies by moving off the host plant [46–49] and must return to the host or find new hosts in order to complete development. Finally, as reported here, caterpillars may defoliate their plants prior to completing development and need to find new plants.

Given a variety of situations in which lepidopteran larvae must locate host foliage, one might expect to find evidence of adaptations for finding host plants. If such adaptations occur, they do not appear to include the antennae. In all lepidopteran larvae, the antennae are very small and, in no species of which we are aware, has pressure to find host plants more efficiently led to the evolution of long antennae. With such small antennae, caterpillars are not thought to be able to detect olfactory stimuli from more than 1 cm away ([50–56] and our study). Likewise, caterpillar vision is crude relative to that of adults [55] and once again, there is no evidence that selective pressure to find hosts has resulted in the evolution of more sophisticated vision. Nevertheless, lepidopteran larvae may possess strategies that improve their chances of finding the next host plant. For instance, arctiid larvae walk especially rapidly for caterpillars [57]. *B*. *philenor* caterpillars too walk rapidly when searching for host plants. Additionally, *B*. *philenor* caterpillars appear able to withstand long periods of starvation, which effectively extends the maximum searching period (D. Papaj, unpubl. obs.). Here, we propose that the elaboration of frontal projections in *B*. *philenor* constitutes an additional adaptation for locating host plants more effectively.

Finally, frontal projections of *B*. *philenor* larvae could possibly have another function not investigated in this study. Also, the function of frontal projections of other lepidopteran larvae may be different from their function in *B*. *philenor*. In particular, the hard and motionless frontal projections of some butterfly larvae may act as weaponry for defence against predators. These possibilities must be investigated in the future.

## Supporting Information

S1 Fig The length of frontal projections relative to their body length in each larval instar. The body length was equalized by changing magnification for all instars. (a) A first instar larva (mean length of first projection: 0.31mm; mean length of body: 4.45mm; length of first projection relative to body length: 0.069; N = 14). (b) A second instar larva (0.97mm; 7.51mm; 0.129; N = 19). (c) A third instar larva (2.39mm; 13.16mm; 0.182; N = 13). (d) A fourth instar larva (5.88mm; 23.98mm; 0.245; N = 15). (e) A last (fifth) instar larva (9.73mm; 40.64mm; 0.239; N = 16).
(TIF)

S2 Fig A larva in the late fourth instar with the base of frontal projections being bound with a fine thread (right), and a larva soon after molting that lost frontal projections without any obvious loss of hemolymph (left). (TIF)

S1 Movie The active movement of frontal projections in *Battus philenor* larvae. (MP4)

S1 Table Data used for statistical analyses and for creating figures. (XLSX)
