# Supplementary figures and images for: Long Frontal Projections Help Battus philenor (Lepidoptera: Papilionidae) Larvae Find Host Plants
Source: PLoS One. 2015 Jul 29;10(7):e0131596. doi: 10.1371/journal.pone.0131596 (PMC4519131; doi:10.1371/journal.pone.0131596)

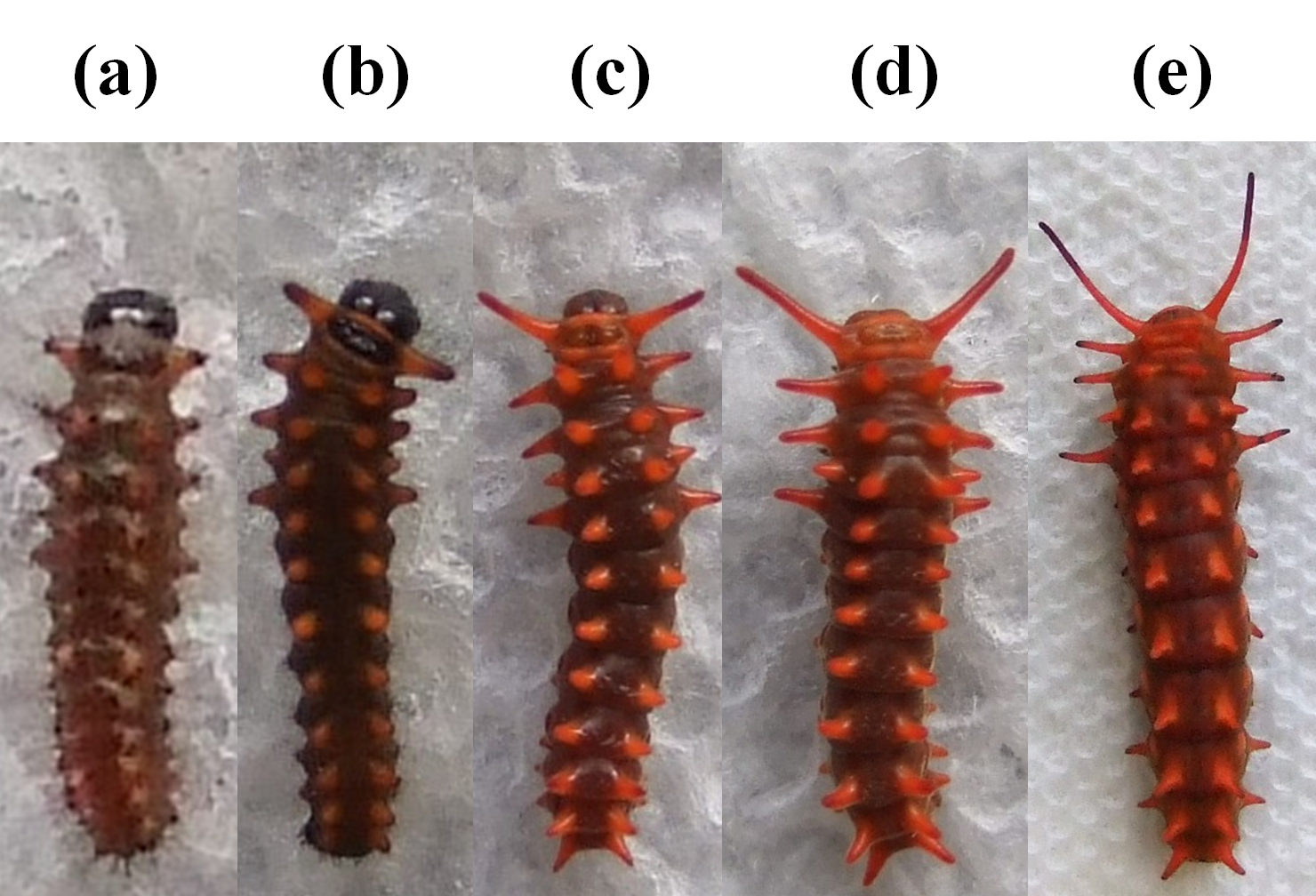

Supplement: S1 Fig — The body length was equalized by changing magnification for all instars. (a) A first instar larva (mean length of first projection: 0.31mm; mean length of body: 4.45mm; length of first projection relative to body length: 0.069; N = 14). (b) A second instar larva (0.97mm; 7.51mm; 0.129; N = 19). (c) A third instar larva (2.39mm; 13.16mm; 0.182; N = 13). (d) A fourth instar larva (5.88mm; 23.98mm; 0.245; N = 15). (e) A last (fifth) instar larva (9.73mm; 40.64mm; 0.239; N = 16). (TIF) [file pone.0131596.s001.tif]

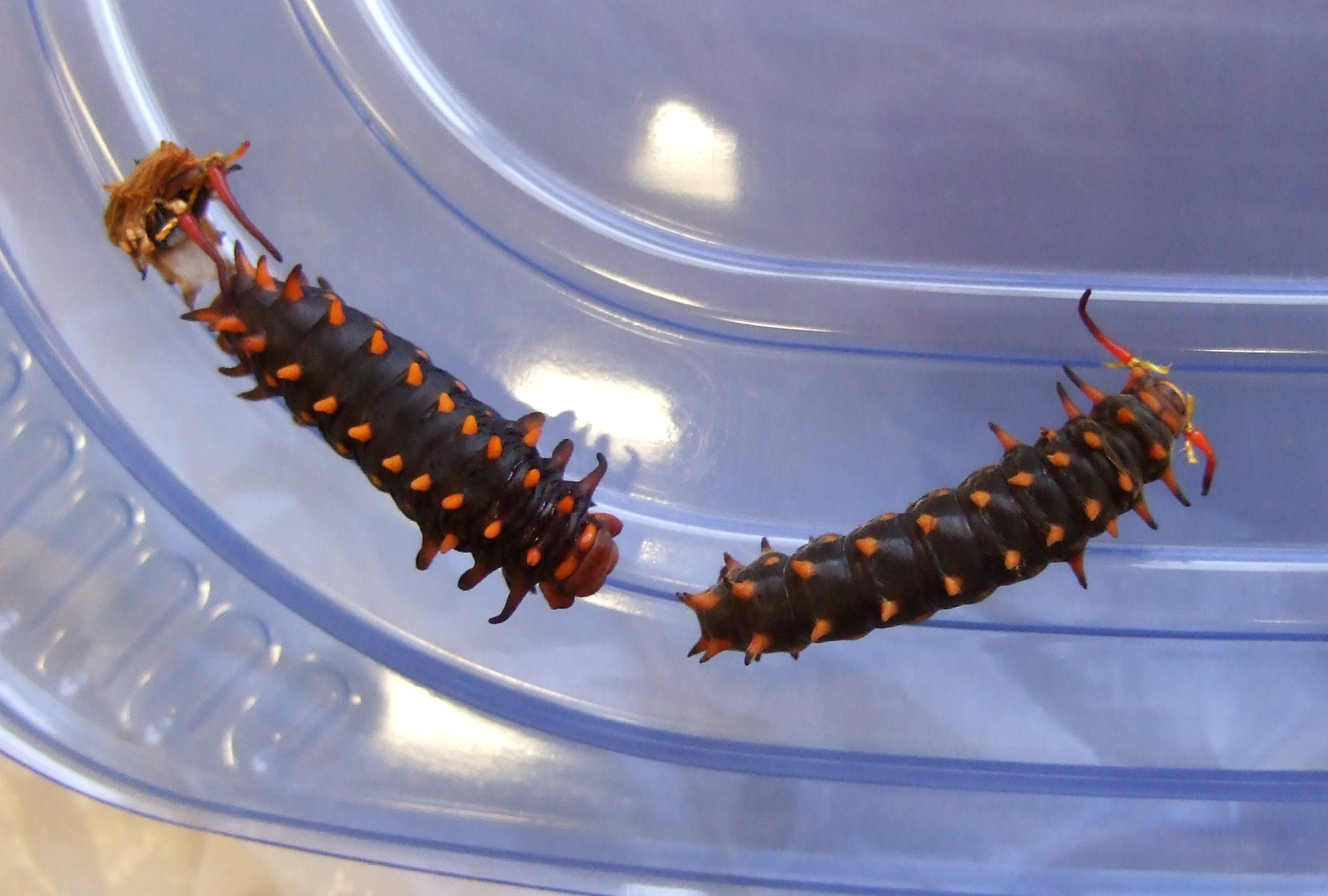

Supplement: S2 Fig — (TIF) [file pone.0131596.s002.tif]
